# Light controls cerebral blood flow in naive animals

**DOI:** 10.1038/ncomms14191

**Published:** 2017-01-31

**Authors:** Ravi L Rungta, Bruno-Félix Osmanski, Davide Boido, Mickael Tanter, Serge Charpak

**Affiliations:** 1 INSERM U1128, Laboratory of Neurophysiology and New Microscopies, Université Paris Descartes, Paris 75006, France; 2 Institut Langevin, Espci Paris, CNRS UMR 7587, INSERM U979, PSL Research University, 17 rue Moreau, Paris 75012, France

## Abstract

Optogenetics is increasingly used to map brain activation using techniques that rely on functional hyperaemia, such as opto-fMRI. Here we test whether light stimulation protocols similar to those commonly used in opto-fMRI or to study neurovascular coupling modulate blood flow in mice that do not express light sensitive proteins. Combining two-photon laser scanning microscopy and ultrafast functional ultrasound imaging, we report that in the naive mouse brain, light per se causes a calcium decrease in arteriolar smooth muscle cells, leading to pronounced vasodilation, without excitation of neurons and astrocytes. This photodilation is reversible, reproducible and energy-dependent, appearing at about 0.5 mJ. These results impose careful consideration on the use of photo-activation in studies involving blood flow regulation, as well as in studies requiring prolonged and repetitive stimulations to correct cellular defects in pathological models. They also suggest that light could be used to locally increase blood flow in a controlled fashion.

## Abstract (web summary)

Combination of optogenetics and BOLD fMRI is routinely used to map neuronal activity upon photostimulation. Here the authors show that light, shone at intensities used in optogenetic studies, dilates vessels and increases blood flow independently of exogenous light-sensitive proteins in the mouse brain.

Over the past 10 years the use of optogenetics to drive genetically distinct populations of brain cells has profoundly increased our understanding of neural circuitry and brain function in health and disease^1^. Optogenetics is now regularly integrated with functional brain mapping techniques such as blood oxygenation level-dependent (BOLD) fMRI in order to generate brain-wide maps of connectivity generated by activation of specific populations of cells^2^,^3^, a technique termed Opto-fMRI. However, the BOLD-fMRI signal is not a direct measure of neuronal activity and reports changes in the concentration of deoxyhemoglobin in vessels. As such, it depends on a complex interplay between functional hyperaemia, oxygen consumption and blood volume^4^. A typical Opto-fMRI experiment involves expression of excitatory and inhibitory light-sensitive proteins, for example channelrhodopsin2 (ChR2) and halorhodopsin variants, in a defined cell population and their activation by brief exposure of light (usually blue for channelrhodopsin2 and yellow for halorhodopsin). Although the use of photoactivation is warranted for the study of cell connectivity *in vitro* and *in vivo*, the side effects of light *in vivo* have not been thoroughly investigated until recently, in particular when trains of light pulses are used^5^,^6^,^7^: In 2013, Christie *et al*. reported that prolonged (30 s) trains of blue light delivery to the naive brain of dead rats caused significant fMRI signals via temperature dependent relaxation changes in T1 and T2* signals^7^. The study thus emphasized the importance of doing control experiments during Opto-fMRI experiments, but also suggested that by testing the absence of fMRI responses upon photoactivation in dead brain tissue, one could eliminate all adverse effects of light^8^. However, the effects of light on T1 and T2* signals may add to an additional and direct effect of light on smooth muscle cells^9^ and blood flow^10^ that would obviously be absent in dead tissue.

Here we combine two-photon laser scanning microscopy (TPLSM) and ultrafast functional ultrasound (fUS) to investigate the effect of light (blue to red) on cerebral blood flow (CBF) in anesthetized naive mice. TPLSM microscopic resolution allows measurements of blood flow in small brain vessels whereas ultrafast functional ultrasound (fUS) is a new macroscopic imaging technique used to measure functional hyperaemia^11^ and resting state blood flow^12^ in large brain regions without contrast agents and with an in plane spatial resolution of 100 by 100 μm and temporal resolution of ∼2 ms. We report that light *per se*, shone through an optic fibre across a chronic cranial window, causes a pronounced pseudo functional hyperaemia, of similar magnitude to a sensory stimulation, in the neocortex and the olfactory bulb. Two-photon Ca^2+^ imaging of GCaMP6f in different transgenic mouse lines reveals that light causes a Ca^2+^ decrease in arteriolar smooth muscle cells that precedes the onset of dilation in the absence of neuronal or astrocyte excitation, suggesting a direct action of light on SMCs.

## Results

### Blue light rapidly increases CBF in the naive mouse brain

**Figure 1:**
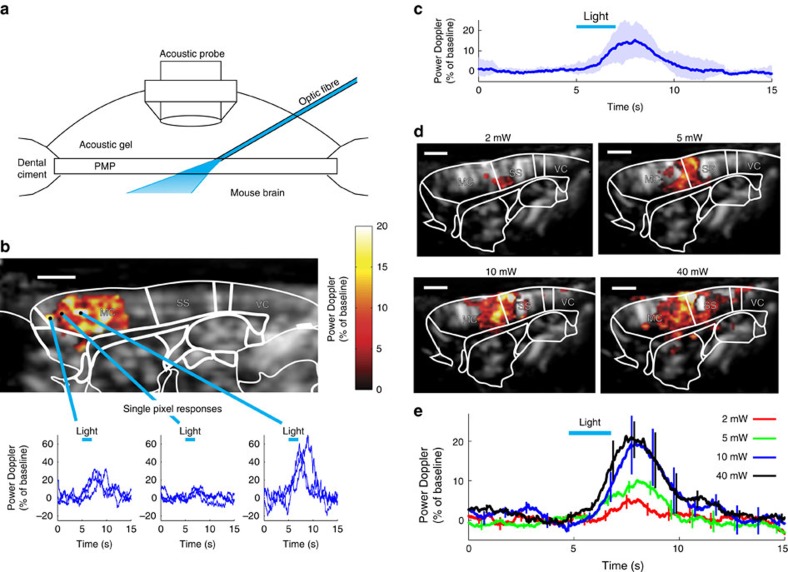
Blue light generates a rapid increase of cerebral blood flow (CBF) in the naive mouse brain. (**a**) Schematic, light emitted by the optic fibre diffuses through a PMP chronic window into the brain. (**b**) A single train of blue light pulses (20 ms, 20 Hz, 5 mW, 2 s) reliably generates a power Doppler signal, detectable at the level of single pixels and indicative of a CBF increase. Top, the activation map is superimposed on a Doppler map. Bottom, single pixel responses vary within the activated region. Three trials are superimposed. (**c**) Average of CBF responses to light in the cortex of three wild type mice. (**d**) CBF responses spread with the stimulus intensity. The region activated by a 2 mW light train (top left, 20 ms, 20 Hz, 2 s) and limited to the border of the somatosensory and motor cortices, enlarges progressively with the intensity shone. (**e**) Averages of CBF responses at different light intensities. Data presented as mean±s.d. All scale bars, 1 mm. fUS in plane resolution =100 × 100 μm^2^.

Trains of blue light of similar duration and magnitude to those used during opto-fMRI experiments (20 ms, 20 Hz, 2 s, 5 mW∼45 mW mm^−2^ at the brain surface, see the calculation in Supplementary methods; Supplementary Fig. 1) were delivered to the brain via an optic fibre, placed directly over the cranial window (Fig. 1a). Each train reliably generated a fUS power Doppler signal, indicative of an increase in CBF and detectable at the level of single pixels (Fig. 1b,c). These hemodynamic responses were dependent on the power of the light delivered as they increased in both magnitude and spatial spread in a dose-dependent manner (Fig. 1d,e). The signals reliably occurred when the power was reduced to 2 mW, measured at the tip of the fibre (Fig. 1d,e), which is an overestimation of what reaches the brain surface (18 mW mm^−2^). These results suggest that blue light itself is capable of increasing CBF, in naive brain tissue.

### Light-evoked CBF increase results from artery dilation

**Figure 2:**
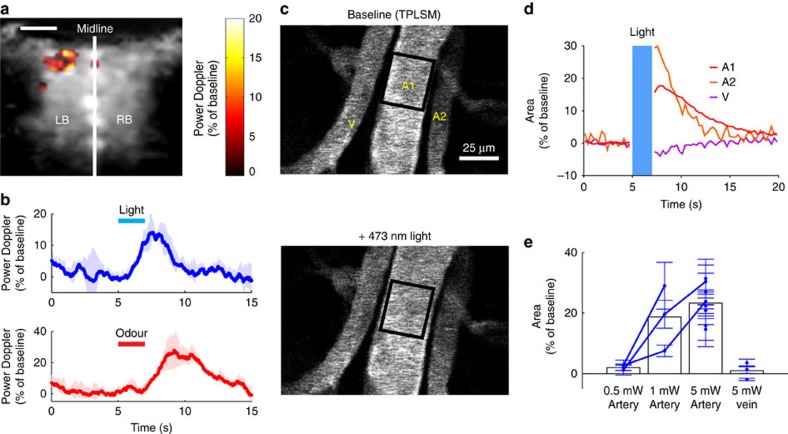
Light-evoked CBF increase results from artery dilation. (**a**) In the olfactory bulb (OB) of the naive mouse, a single train of blue light (20 ms, 20 Hz, 5 mW, 2 s) generates a focal increase of the power Doppler signal. Scale bar, 1 mm. In plane resolution: 100 × 100 μm^2^. (**b**) Averages of CBF responses to light and odour (ethyl tiglate). (**c**) Light causes a dilation of large arterioles at the dorsal surface of the OB. Vessels were labelled with Texas red and imaged with a two-photon microscope. Light dilated both A1 and A2 arteries but did not affect the vein V. (**d**) Quantification of the experiment in (**c**). (**e**) Dilation is detectable above a light intensity of 1 mW. Data points correspond to mean value of 3–5 trials±s.d. in individual mice.

We next investigated the vascular site (arterioles, veins and capillaries) at which light could generate fUS signals. We performed these experiments in the olfactory bulb due to its well defined circuitry, and the tight relationship between synaptic activation, functional hyperaemia and BOLD fMRI responses within the glomerular layer^13^,^14^,^15^,^16^,^17^. First we verified, using fUS, that blue light similarly caused consistent macroscopic increases in CBF. These increases were within the range of odour-evoked responses (Fig. 2a,b). We next used TPLSM to determine the location of these hemodynamic responses microscopically. Unlike fUS, imaging the vasculature with TPLSM required addition of a contrast agent injected intravenously (IV). Texas Red (70kDa) was preferred to FITC, due to the non-overlapping excitation spectrum with 473nm light and therefore avoidance of the ROS-mediated blockade of propagated dilation along arterioles^18^,^19^. Surface arterioles consistently dilated following stimulation with blue light (Fig. 2c–e), while the diameter of veins within the same field of view were not affected, suggesting that blue light evoked a relaxation of vascular smooth muscle cells (SMCs). The threshold of the arteriole dilation was in the range of 0.5–1 mW (Fig. 2e), corresponding to a mean value between 12.5 and 25 mW mm^−2^ (Supplementary methods; Supplementary Fig. 1). Although blue light is most commonly used in optogenetic experiments, yellow-green light is used to activate Halorhodopsin and Archaerhodopsin inhibitory opsins, and additional red-shifted ChR variants also now exist^20^,^21^,^22^,^23^. We therefore tested the dilatory response to a spectrum of wavelengths. Trains of yellow-green, orange and red light (561 nm, 594nm and 638nm) also dilated arterioles, but slightly less efficiently, and with the dilation decreasing in magnitude as the wavelength was increased (Supplementary Fig. 2).

### Light does not increase neuron or astrocyte Ca^2+^

**Figure 3:**
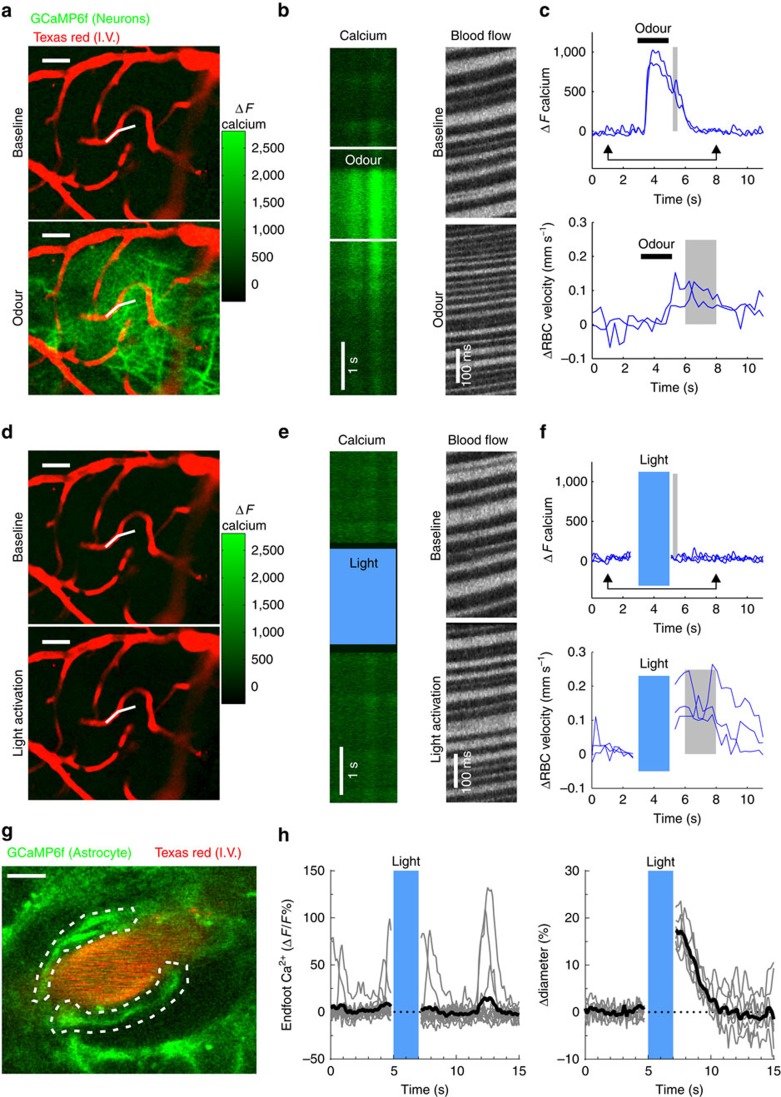
Light increases CBF independently of neuronal or astrocyte Ca^2+^ dependent mechanisms. (**a**) Odour causes a large calcium increase in the glomerular layer of a mouse expressing GCaMP6f under the Thy1 promoter. Top, Fluorescence increases robustly in the dendritic tufts of mitral cells during odour. Images were selected from a frame scan acquisition. The broken line in white indicates the two segments used in linescan acquisition mode to measure calcium and red blood cell (RBC) velocity in **b**–**f**. (**b**,**c**) Odour generates a calcium increase in the neuropil that precedes the increase in RBC velocity by more than a second. The calcium raw data shown in (**b**) corresponds to the acquisition comprised between the two arrows in (**c**). The RBC raw flow data shown in (**b**) were selected from baseline and following odour. (**d**-**f**) Light increases RBC velocity without activating neurons. All grey areas illustrate the time periods used to quantify the effects of odours and light (see main text). Scale bar in (**a**,**d**) is 25 μm. (**g**) An arteriole whose lumen is labelled with Texas red and that is surrounded by astrocyte end-feet expressing GCaMP6f under the connexin 30 promoter. Dashed lines outline endfoot ROIs plotted in (**h**). Scale, 5 μm. (**h**) Light dilates the vessel (right) without affecting the spontaneous calcium signals nor the steady state calcium level in the astrocyte end-feet(left). Grey traces show single trials, black trace shows mean of trials.

Light *per se*, delivered with different excitation modes (one and two-photon excitation) and at various wavelengths has been shown to directly activate neurons^24^,^25^,^26^,^27^, and thus it is possible that trains of light triggered the release of vasoactive compounds normally released during neurovascular coupling. To test the possibility that light directly activated neurons, we delivered trains of blue light through an optic fibre across a chronic cranial window of Thy1-GCaMP6f (GP5.11) mice. These mice strongly express GCaMP6f in mitral cells^28^, the principal neurons of the olfactory bulb. The tufts of mitral cells fill individual glomeruli (located 40–50 μm below the brain surface) that are specifically and potently activated by odours^29^,^30^. We first identified glomeruli responsive to specific odours. Figure 3a shows a glomerulus, comprising the dendritic tufts of mitral cells in which calcium increased upon a 2 s inhalation of Ethyl tiglate. A broken line scan, acquired within a capillary and extending into the neuropil showed that odour stimulation triggered an increase in the post-synaptic calcium concentration that preceded an increase in capillary red blood cell (RBC) velocity (Fig. 3b,c), typical of neurovascular coupling in the glomerular structure. In contrast (Fig. 3d-f), blue light consistently evoked an increase in RBC velocity in the same capillary, but in the absence of an observable elevation in post-synaptic calcium. This light-triggered increase in blood velocity was reversible and reproducible (Odour: mean Δ*F*=300±200, mean ΔRBC velocity (%): 40±20; Light: mean Δ*F*=0±8, mean ΔRBC velocity (%): 30±20, mean±s.d., *n*=3 mice). These experiments demonstrate that blue light does not activate neurons and conversely suggests that it could directly act on other cells involved in neurovascular coupling or in the regulation of vascular tone, i.e. astrocytes, pericytes or SMCs. To test whether astrocyte Ca^2+^ elevations were evoking arteriole dilations, we performed a separate set of experiments in mice expressing GCaMP6f under control of the connexin 30 promoter. This mouse line showed high expression of GCaMP6 in astrocyte end-feet enwrapping arterioles (Fig. 3g). End-feet showed slow spontaneous calcium transients. Light dilated parenchymal arterioles without increasing the steady state level of calcium and the slow calcium transients (Fig. 3h) (light trains: arteriole dilation (%): 10±5, Δ*F*/*F* (%): –4±1, mean±s.d., *n*=4 arterioles, 2 mice). These experiments suggest that the photodilation is not mediated by activation of neuronal and/or astrocyte Ca^2+^-dependent neurovascular coupling pathways.

### Light-triggered dilations are caused by a decrease in SMC calcium

**Figure 4:**
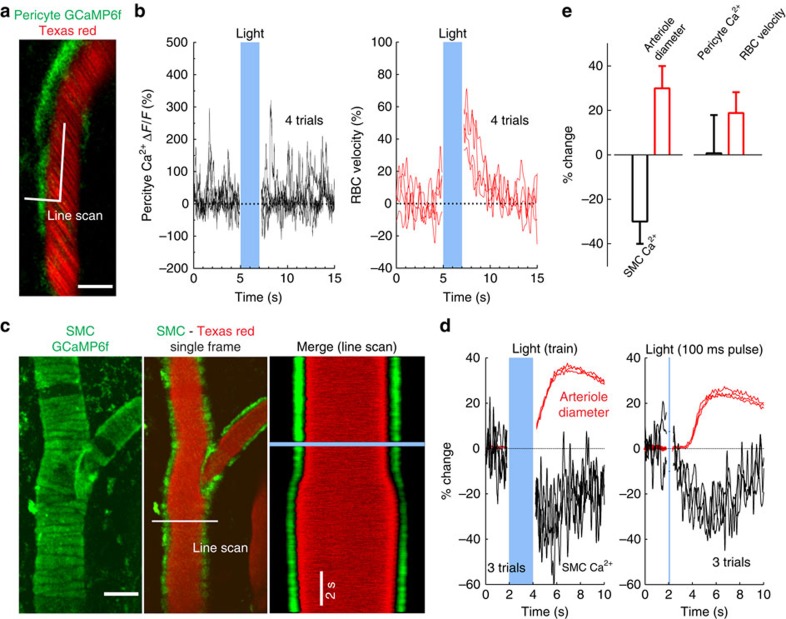
Light triggers dilation via a decrease in SMC calcium. (**a**,**b**) A glomerulus layer capillary, whose lumen is labelled with Texas red and contacted by the process of a longitudinal-type pericyte expressing GCaMP6f. A broken line scan acquisition to simultaneously measure pericyte calcium and RBC velocity. Scale bar, 3 μm. (b) Spontaneous pericyte Ca^2+^ transients or steady calcium are not affected by consecutive light trains (5 mW, 20 ms, 20 Hz, 2 s), whereas RBC velocity is consistently increased (right). (**c**,**d**) An arteriole in which the smooth muscle cell (SMC) wall shows typical stripe patterns of GCaMP6f expression, and in which the lumen is labelled with Texas red. A transversal linescan acquisition allows simultaneous recording of SMC calcium and vessel diameter. Light lowers calcium in the SMC wall and dilates the vessel. Scale bar, 30 μm (**d**) Left, the reversible responses to three consecutive trains (5 mW, 20 ms, 20 Hz, 2 s) are superimposed. Right, single 100 ms pulses (5 mW) reveal that the decrease in SMC calcium precedes the arteriole dilation (displayed in (**c**) on right). (**e**) Summarized data: Left, SMC Ca^2+^ and arteriole diameter (*n*=3 arterioles, 3 mice). Right, glomerulus pericyte Ca^2+^ and RBC velocity (*n*=6 capillaries, 3 mice). Data presented as mean±s.d.

We then investigated whether light activated pericytes and/or SMCs in adult mice expressing GCaMP6f under control of the NG2 promoter^31^,^32^. In these mice, pericytes, SMCs and oligodendrocytes expressing GCaMP6f can be easily distinguished on the basis of their morphology. Glomerular capillaries are covered by longitudinal-type pericytes, expressing GCaMP6f in their somata and processes, and in which we recorded spontaneous calcium transients (Fig. 4a,b). As was observed in astrocytes, light did not affect the steady state calcium level or the calcium transients in these pericytes, while it caused an increase in RBC velocity (Fig. 4b,e) (Δ*F*/*F* (%): 1+/−17, ΔRBC velocity (%): 19±9, mean±s.d., *n*=6 capillaries, 3 mice). Glomerular capillaries had a mean diameter of 3.3±0.5 μm (mean±s.d.) and no attempt was made to investigate whether light caused a minute passive change in capillary diameter (1–2%), as reported in the retina upon visual stimulation^33^. These results suggest that light acts up-stream and directly relaxes SMCs, thereby increasing blood flow in the capillary bed. Figure 4c illustrates that GCaMP6f expression enabled us to identify SMCs enwrapping arterioles (Fig. 4c). Light reduced intracellular calcium in SMCs, an effect that preceded the vessel dilation (Fig. 4d,e) (light trains: arteriole dilation (%): 30±10, ΔF/F (%): −30±10; single light pulse (5 mW, 100 ms): arteriole dilation (%): 14±9, Δ*F*/*F* (%): 20±10, mean±s.d., *n*=3 mice). Finally, to eliminate the possibility that light activated additional calcium-independent mechanisms in astrocytes or neurons that would indirectly cause SMC dilation, we tested whether the photodilation occurred in other peripheral organs. Supplementary Fig. 3 shows that light, shone at similar intensities, also increased blood flow in the kidney. We conclude that light dilates arterioles by directly relaxing cerebral SMCs independently of the release of vasoactive compounds from neurons or astrocytes.

### Light-triggered dilations are blocked by isoflurane anaesthesia

**Figure 5:**
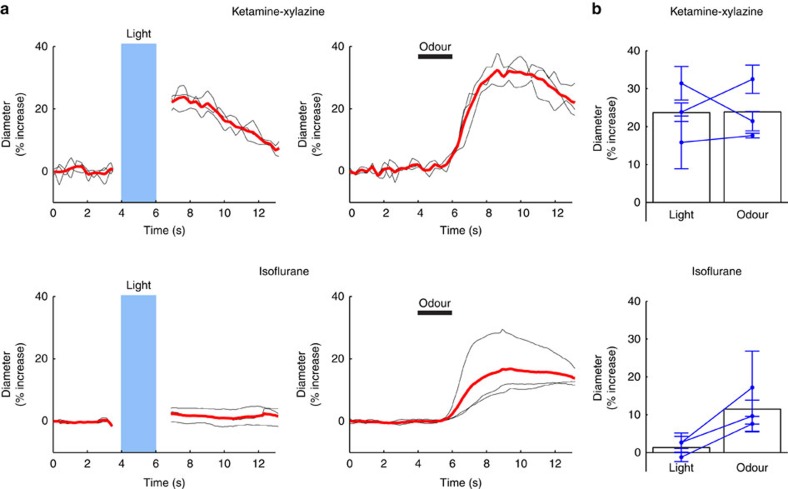
Isoflurane blocks light-triggered dilations. (**a**) The same vessel that dilated to light under ketamine-xylazine anaesthesia (top left) no longer dilated to light when the mouse was anesthetized with isoflurane (bottom left). Right, postive interleaved controls show response to odour indicating that neurovascular coupling is maintained, although smaller. Black traces: single trials from the same vessel. Red trace: mean. (**b**) Summarized data (3 mice). Each point represents mean±s.d. for individual mice.

Photodilation occurs at an energy deposit threshold of about 0.4−0.8 mJ, a value that generates little heat, that is, 0.07 °C (see Supplementary Fig. 2, and discussion), in contrast with the larger temperature changes required to induce BOLD signals in dead animals^7^. Moreover, it is surprising that it has not been reported in several opto-fMRI studies (for example, refs 2, 3, 34) that correctly controlled for the absence of BOLD signal generation in control animals (wild-type or ChR2 negative) in response to supra-threshold trains of light. We thus investigated whether the key difference in these studies resulted from the use of isofluorane anaesthesia. Volatile anaesthetics such as isoflurane, have multiple protein targets, are potent cerebral vasodilators and there exists a narrow range of isoflurane concentrations (<1.5–2%) under which neurovascular coupling is preserved^35^,^36^,^37^. We therefore tested whether isoflurane altered photodilation at an anaesthesia level under which responses to odour were conserved (0.7–1.1% isoflurane, arriving at the nose cone, see Methods). Experiments on the same vessels were interleaved between light and odour stimulations and additionally, the same vessels were imaged on different days with identical placement of the optic fibre to compare the response under isoflurane and ketamine-xylazine anaesthesia. Surprisingly, arterioles which reproducibly dilated to blue light under ketamine-xylazine anaesthesia did not dilate under isoflurane (Fig. 5) (Arteriole dilation (%) under Ketamine-xylazine, odour: 24±8, light: 24±8; Arteriole dilation (%) under isoflurane, odour: 12±5, light: 1±2, mean±s.d., *n*=3 mice). Importantly, odour-evoked arteriole dilations were maintained, although smaller, under isoflurane anaesthesia.

## Discussion

This work shows that light per se, delivered in trains and at intensities commonly used to trigger functional hyperaemia and/or fMRI signals in rodents, decreases SMC calcium, either directly or via endothelial cells, leading to dilation of arterioles. The effect of light is in the same range of amplitude as that evoked by sensory stimulation (ΔRBC velocity (%) with light and odour stimulation, respectively: 30±20 and 40±20, paired capillaries, *n*=3 mice), and will thus affect the signal size and threshold detected in opto-fMRI experiments. Although the threshold for photodilation required more energy than required to activate ChR2 expressing neurons (single pulse, a few ms), it was lower than what is often reported for opto-fMRI experiments. Photo-activation of light-sensitive proteins thus remains a valid tool to study the role of specific neuronal subtypes in signal processing. However, careful consideration must be taken for its use in studies that rely upon blood flow regulation, e.g. neurovascular coupling or BOLD and cerebral blood volume fMRI. This also complicates the interpretation of studies using transgenic mice expressing ChR2 in SMCs, in which light will have two opposite actions: the photoactivation of ChR2 depolarizing the cells and raising their intracellular calcium leading to constriction, and the direct effect of light decreasing calcium leading to dilation.

The result that isoflurane anaesthesia blocked the photodilation clarifies why opto-fMRI studies in which isoflurane was used show no BOLD signal in their light control experiments. However, it also indicates that both investigation and control experiments must be performed under the exact same experimental conditions. It also advocates in favour of α2-adreno-receptor agonists such as Medetomidine for sedation protocols in fMRI studies^38^,^39^. It should be stressed that in well-designed studies where light stimulation protocols are delivered at energy below the threshold of photodilation (for example,^40^), the photoactivation of a specific protein in a given cell type may be accurately related to a resulting change in CBF.

Our Ca^2+^ imaging experiments performed in transgenic mice expressing GCaMP6 in various cell types of the neurovascular unit provide some substantial insight about the cell types involved: the lack of Ca^2+^ elevations in astrocyte endfeet and mitral cell dendritic tufts rules out that light stimulation recruits ‘classical' calcium-dependent neurovascular coupling mechanisms. Whereas the Ca^2+^ imaging did not rule out the possibility that other subtypes of neurons, for example, interneurons, were activated or that Ca^2+^-independent mechanisms were recruited, the fact that light also dilated arterioles in the kidney, confirms that neurons or glial cells are not players in this effect. Our results are highly suggestive that light leads to Ca^2+^ efflux from SMCs by acting directly on the SMC itself, or indirectly via the endothelium.

The molecular mechanism and signalling pathway involved in photodilation remains unclear: it is sensitive to isoflurane, and is located upstream of actin/myosin interactions in the signalling pathway, as a decrease in SMC Ca^2+^ precedes the dilation. The obvious mechanism that could trigger dilation is heat. Stujenske *et al*.^6^ recently measured and modelled heat generated in brain tissue through an optic fibre. We implemented our fibre characteristics and the light protocols in their model (see Methods) and calculated that our common protocol (20 ms, 20 Hz, 2 s, 5 mW) should increase the local temperature by 0.38 °C, consistent with experimentally measured temperature changes^6^,^7^. However, the energy threshold at which dilation appears (<1 mW, 20 ms pulses, 20 Hz, 2 s; <0.8 mJ) should cause a temperature increase of only 0.07 °C. Such value is within the range of natural fluctuations observed in the awake brain^41^, which are not tightly correlated to blood flow changes.

The excitation spectrum of photodilation shows a decrease in magnitude with increasing wavelength (Supplementary Fig. 2). The persistence of a dilation at longer wavelengths used to activate red-shifted rhodopsins stresses that controls must be maintained in all cases. Such a wavelength dependency matches what would be expected from a heat-dependent mechanism. Therefore, even though our estimated temperature shifts are extremely small, we cannot rule out the possibility that heat contributes to the effect and that one of the numerous proteins, which participate in endothelial and smooth muscle cell membrane potential regulation, such as some TRP channels, voltage-gated potassium channels or metabolite transporters^42^,^43^,^44^,^45^,^46^,^47^,^48^,^49^,^50^, is exquisitely heat sensitive. Conversely, the fact that photodilation decreases at longer wavelengths also fits with light absorbance by a specific protein involved in vascular tone regulation, independently of heat. Whatever the mechanism involved, the reversibility and reproducibility of the photodilation, indicate that it could be used as a simple technical means to increase blood flow in a controlled fashion in pathological tissues requiring more oxygen.

## Methods

### Animal preparation and surgery

All animal care and experimentation was performed in accordance with the INSERM Animal Care and Use Committee guidelines (protocol numbers CEEA34.SC.122.12 and CEEA34.SC.123.12). Adult mice (2–6 months old, 20–35 g, both males and female, housed in 12-h light-dark cycle) were used in this study. Mice strains were obtained from the following suppliers; *C57BL/6*, Janvier Labs; *Thy1-GCaMP6f (GP5.11)*, Jackson laboratory, *Ai95(RCL-GCaMP6f)* were donated from Hongkui Zeng (Allan Institute), *NG2-CreERT2* were donated from Frank Kirchhoff (ULM University). All mice were bread on a *C57BL/6* background. To generate mice with conditional GCaMP6f expression in mural cells, *NG2-CreERT2* were crossed with *Ai95(RCL-GCaMP6f)* mice. *NG2-CreERT2, Ai95(RCL-GCaMP6f)* double transgenic mice were administered 2 mg of tamoxifen for 1–3 consecutive days and imaging was done 2–8 weeks later. The same strategy was used to generate mice with specific expression in astrocytes except that a *Connexin30-CreERT2* (donated from Frank W Pfrieger), was crossed with a *Ai95(RCL-GCaMP6f)* mouse.

Chronic craniotomies were performed as previously described^51^. In brief, mice were initially anesthetized with an intraperitoneal (IP) bolus of ketamine-xylazine (100 mg kg^−1^ and 10 mg kg^−1^ body mass, respectively). Further 10–20% of the same mixture was injected IP as necessary to maintain surgical plane anaesthesia. During surgery, the mice breathed a mixture of air and supplementary oxygen and the body temperature was monitored by a rectal probe and maintained at ∼36.5 °C by a feedback-controlled heating pad. A craniotomy was performed with a dental drill and care taken not to apply pressure to the bone and the area was regularly flushed with cool aqueous buffer solution to avoid damage or heating of the underlying tissue. Either a cover glass (100 μm thick) or Polymethylpentene (PMP) (250 μm thick) was used for the window and sealed in place with photopolymerizable dental cement, which was also used to form a head-cap in which a titanium head-bar was also embedded.

The following veterinarian medications were used pre-, during and post-surgery; the anti-inflammatory, dexamethasone (Dexazone, 5 mg kg^−1^ body mass), administered once daily by subcutaneous injection pre-surgically and one day post-surgery; the analgesic, buprenorphine (Buprecare, 0.1 mg kg^−1^ body mass), administered by subcutaneous injection after the surgery and the following post-surgical day if necessary; The antibiotic enrofloxacine (Baytril, 5 mg kg^−1^ body mass), administered by subcutaneous injection pre-surgically and for two days post-surgery. Mice were permitted to recover for at least 1 week, before the experimental sessions began.

For experiments mice were anesthetized with ketamine-xylazine (100 mg and 10 mg kg^−1^ body mass, respectively) injected IP. Experiments were performed within 20–120 min following injection of anaesthetics. Depth of anaesthesia was monitored with breathing rate (2–3 Hz) recorded by a pneumogram transducer (Biopac Systems) and toe pinch reflex. Body temperature was maintained at ∼36.5 °C using a heating pad. In a subset of experiments performed under isoflurane anaesthesia (Fig. 5), mice were induced with 3% isoflurane for 1.5 min. For experiments, total flow to the nose cone was (1 L of air/min); (700 ml air/min) from the isoflurane apparatus set between 1 and 1.5% isoflurane, and an additional (300 ml air per min) from the olfactometer to deliver odour, thereby diluting the isoflurane concentration arriving at the nose cone to 0.7–1.1%.

For acute kidney experiments, the same anaesthetics were used as for the chronic window implantation. A ∼2 cm incision was made on the skin to expose the kidney. A plastic palette was gently inserted in between the kidney and the diaphragm to prevent movement artefacts during the fUS experiments. The optic fibre was placed directly on the surface of the kidney and ultrasound gel was gently placed between the ultrasound probe and the kidney.

### Imaging the mouse brain with fUS

Following anaesthesia the cranial PMP window was rinsed with sterile saline and 1 cm^3^ of ultrasound coupling gel was placed between the window and the linear ultrasound probe (15 MHz central frequency, 128 elements; Vermon; Tours, France). The transducer was connected to an ultrafast ultrasound scanner (AixplorerT.M, SuperSonic Imagine; Aix-en-Provence, France). Programming of custom transmit/receive ultrasound sequences was done in Matlab (MathWorks; Natick, Massachusetts, USA), using software-based architecture of the scanner.

### Ultrasound sequences

The concept of ultrafast Doppler relies on compounded plane-wave transmissions^52^. The mouse brain was insonified with a succession of ultrasound plane waves and the backscattered echoes were recorded and beamformed to produce an echographic image for each transmission. Although the frame rate of ultrafast ultrasound can reach more than 10 kHz, a 500 Hz frame rate was used as it allows correct sampling of the ultrasound signals backscattered by the red blood cells without aliasing in the mouse brain^53^. To increase the SNR of each echographic image taken at 500 Hz, the echographic images were compounded by transmitting several tilted plane waves and added their backscattered echoes. The compounded sequence resulted in enhanced echographic images, thereby increasing the sensitivity of the Doppler measurement^54^. In this study, the ultrasound sequence consisted of transmitting eleven different tilted plane waves (−10, −8, −6, −4, −2, 0, 2, 4, 6, 8, 10° tilted angle) with a 5,500 Hz pulse repetition frequency (PRF). The backscattered echoes were added to produce enhanced echographic images at a 500 Hz frame rate.

### Power Doppler data treatment

As the backscattered signals from the mouse brain are composed of both tissue and blood signals, the following steps were performed to remove signals from the tissue. First, a singular value decomposition (SVD) was applied on the stack of the fUS images and the largest Eigenvalues were eliminated to filter out the slowest variations in the Power Doppler signal which represented the tissue signal^55^. Next, the backscattered signals were filtered with a fourth order Butterworth high-pass filter with a cut-off frequency of 50 Hz to further remove any tissue or motion artefacts. The Doppler signal of each spatial pixel was obtained by the incoherent temporal mean of the blood signal. The increase in Power Doppler signal (proportional to the cerebral blood volume^54^) evoked by the light stimulations was measured in each pixel, which were 100 × 100 μm^2^ in plane size with a slice thickness of 200 μm.

### Building activation maps

Activation maps were made using average power Doppler signals from 3 to 5 trials. Activated pixels were found using a Pearson correlation coefficient r between the local power Doppler temporal signal computed from each spatial pixel of the fUS acquisition and a temporal binary step pattern from 0 to 1 for a duration of 3 and 4 s (for the brain and the kidney respectively) starting 1.5 s after the start of the light train stimulus. Activations were considered significant for a correlation *r*>2σ, where σ is the spatial standard deviation of the correlation map. Once activated areas were found, the activation maps were displayed as the percentage increase from baseline of the power Doppler signal, measured for 1 s and 2 s (for the brain and the kidney respectively) following the end of the light train. Power Doppler time course variations for individual pixels are shown in Fig. 1b. Averaged time course plots were calculated by averaging the temporal signal of the 10 most activated pixels contained within the activated region (*r*>2σ). In a few cases (particularly in the kidney) 5–10 pixels were significantly activated. Signals were never observed using the same criteria in the absence of light.

### Two-photon laser scanning microscopy

Imaging was performed using a femtosecond laser (Mai Tai eHP; SpectraPhysics) with a dispersion compensation module (Deepsee; Spectraphysics) emitted ∼70-fs pulses at 80MHz. Laser power was attenuated by an acoustic optical modulator (AA Optoelectronic, MT110-B50-A1.5-IR-Hk). XY scanning was performed with Galvanometric scanner (GS) mirrors (VM500; GSI Lumonics). GCaMP6 and Texas Red were excited at 920nm. Emitted light was collected with a 40X/0.8NA objective (Leica) and was sent to a pair of lenses, coupled into a 2-mm diameter core polymethyl methacrylate optical fibre as previously described^56^. Collected light was split using a dichroic mirror at 580 nm and the signals were each detected with a dedicated GaAsP photomultiplier tube (Hamamatsu) after passing through an appropriate emission filter (GCaMP6: 525 nm, 50 nm bp; Texas Red: 620 nm, 60 nm bp). Customized Labview software was used to control imaging paramaters. A mechanical shutter was placed directly before the 580 nm dichroic mirror to shield the PMTs during the photostimulation period. The laser light was blocked for a few additional milliseconds before and after the photostimulation period. Texas Red dextran (70 kDa, Molecular Probes) was administered intravenously by retro-orbital or tail vein injection. Analysis of vessel dilations represents maximum absolute value compared with baseline. Smooth muscle cell calcium decreases were averaged over 1 s surrounding the maximum absolute value compared with baseline. For mean values in pericyte and astrocyte experiments, Ca^2+^ measurements were averaged during 2 s after light delivery, relative to the 2 s time period before light.

### Light and Sensory stimulation

Optical stimulation, was performed with a 473 nm laser (Coblot MLD, Sweden), a dual laser 488 nm/561 nm (Oxxius, France), or 594 nm and 638 nm lasers (Oxxius, France) with FC/PC coupler to deliver the light pulse. The light pulse was triggered through an analogue module to deliver optical stimulations: Trains (20 ms, 20 Hz, 2 s duration) or single continuous pulses (100 ms duration). The multimode optical fibre was 62.5 μm (GIF625; ThorLabs, Germany). The light power delivered from the fibre tip was calibrated using optical power metres (Gentec-eo, Canada) and was measured during continuous mode. For spectrum comparison of 473, 594 and 638 nm lasers, the input to the optical fibre was manually moved between lasers without moving the position of the tip. Un-connecting and reconnecting the fibre from the laser resulted in power changes of <5%, measured at the fibre tip. Calculations of power in terms of mW mm^−2^ can be found in Supplementary methods; Supplementary Fig. 1.

Odour stimulation was performed with a custom built olfactometer controlled with customized Labview software. Pure air was constantly delivered to the mouse nose and a valve switched the flow from air to an odour-air mixture (800 ml min^−1^) for a 2 s stimulation. The pressure of the air line and the odour line were measured and balanced before starting the experiment. Clean air or odour mixture was supplemented with 200 ml min^−1^ O_2_.

Paired experiments were interleaved, no randomization or blinding was used. No statistical methods were used to predetermine sample sizes. No mice were excluded from analysis.

### Modelling of heat generation

Simulations to estimate of the maximal local temperature increase upon our light stimulation protocols were performed using the model by Stujenske *et al*.^6^. Matlab scripts were downloaded from: http://www.sciencedirect.com/science/article/pii/S2211124715006488. We first modelled the light distribution in space with the Monte Carlo algorithm (MonteCarloLight and LightHeatPlotter Matlab scripts), using Johansson's model (default). Calculations were made based on the features of the optic fibre used for all our experiments (GIF625; ThorLabs, Germany); a fibre radius of 31 μm and NA=0.275, and stimulation protocols (40 pulses of 20 ms at 20 Hz, laser power of 5 or 1 mW at the end of the fibre). Temperature changes were modelled for all wavelengths used (473, 488, 561, 594 and 638 nm) without changing any of the default values. To obtain temperature increases caused in the brain tissue by our stimulation pulses we used the HeatDiffusionLight script and plotted the time evolution of the temperature (time versus depth plot). The maximum increase of temperature from these plots for each wavelength was plotted in Supplementary figure 2. Note that the values represent overestimates of the maximum temperature change because the model is based on an optic fibre inserted vertically in the brain while we had an oblique optic fibre placed at distance and a piece of coverslip of 100 μm between the fibre tip and the brain. Light attenuation across the coverslip is negligible.

### Data availability statement

The data that support the findings of this study are available from the corresponding author on reasonable request.

## Additional information

**How to cite this article:** Rungta, R. L. *et al*. Light controls cerebral blood flow in naive animals. *Nat. Commun.* **8,** 14191 doi: 10.1038/ncomms14191 (2017).

**Publisher's note:** Springer Nature remains neutral with regard to jurisdictional claims in published maps and institutional affiliations.

## Supplementary Material

Supplementary Information Supplementary Figures and Supplementary Methods.
