# Supplementary material for: Light controls cerebral blood flow in naive animals
Source: Nat Commun. 2017 Jan 31;8:14191. doi: 10.1038/ncomms14191 (PMC5290324; doi:10.1038/ncomms14191)
Supplement: Supplementary Information — Supplementary Figures and Supplementary Methods. [file ncomms14191-s1.pdf]

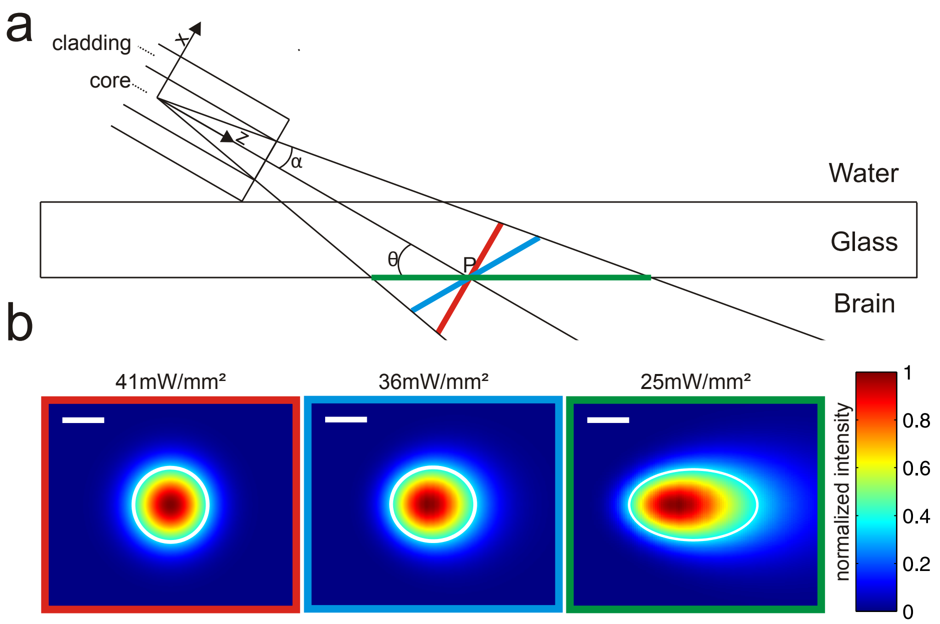

Supplementary Figure 1.
Estimation of the light power and intensity profile reaching the brain.

(**a**) The optic fiber (NA=0.275) makes an angle θ=30° with the cranial window made in glass (100µm thick) or PMP (250 µm). (**b)** Computation of the intensity in three planes (red line, for θ=90°, blue line for θ=45° and green line for θ=30°). Note that the light beam enlarges when θ decreases and the intensity profile becomes elliptic at the surface of the brain (see **Supplementary methods 1** for the theory analysis and the numerical calculations). Scale bar: 100µm.

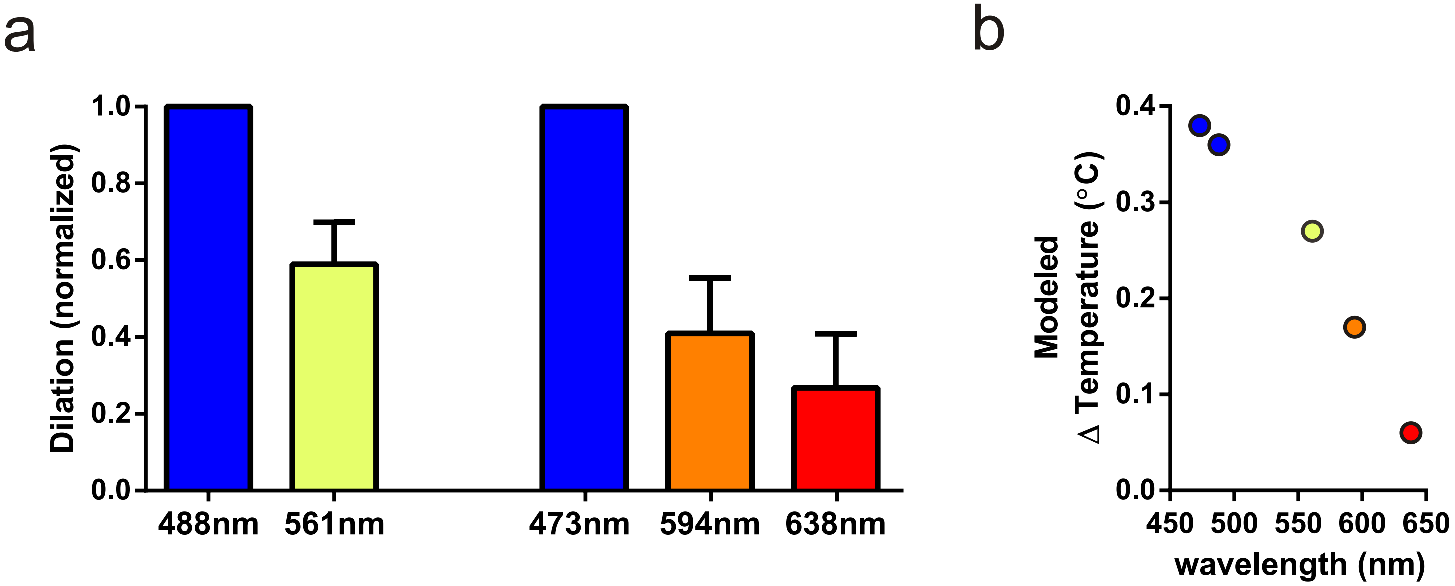

Supplementary Figure 2: Spectrum of photodilation

(**a**) Pial vessels were photo-stimulated by trains (5 mW, 20 ms pulses, 20Hz, 2 s duration) of light at different wavelengths. Photodilation decreases in magnitude at longer wavelengths. Left: a dual laser (488 nm and 561 nm), allowed collimating the two beams in the same fiber at the same intensity. (3 vessels, 3 mice) Right: 3 lasers of different wavelengths were alternated without moving the placement of the fiber in relation to the brain (4 vessels, 3 mice). Displayed as mean ± SD. (**b**) Values of maximum local temperatures generated at the fibre tip, as calculated by inputting our standard protocol values (5 mW, 20 ms pulses, 20Hz, 2 s duration) in the open source matlab code program from Stujenske et al.[6]. Temperature elevations decrease at longer wavelengths.

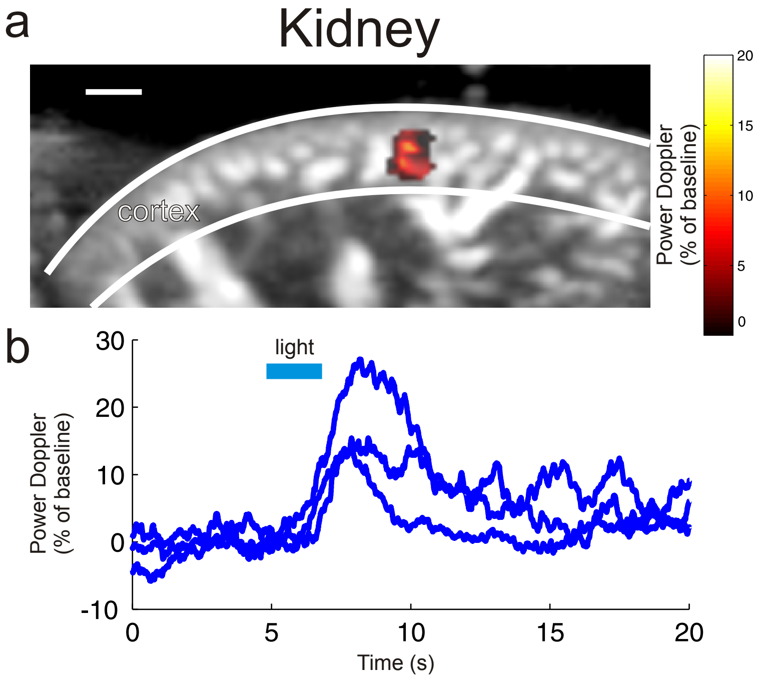

Supplementary Figure 3.
Blue light generates a rapid increase of blood flow in the kidney of a naive anesthetized mouse.

(**a**) A single train of blue light pulses (20 ms, 20 Hz, 5 mW 2 s) reliably generates a power Doppler signal in the cortex of the kidney. Scale bar: 1mm. fUS in plane resolution: 100x100 µm. (**b**) Time course of the response in 3 individual mice.

Supplementary Methods

Calculation of average optical power

The goal of this section was to calculate the average optical intensity given by an optic fiber at the interface between the glass and the brain. The experimental set up is described in **Supplementary Figure 1a.**

Theoretical Analysis:

The first step was to model analytically the light beam using a Gaussian model. We neglected the added diffraction due to the water (the optical index of water (1.33) is lower than glass (1.5) or PMP (1.46)) and made the approximation that the light emitted by the fiber enters directly into the glass.

The light emitted by the fiber was modeled as a cone with an angle α.

$$\alpha = \sin^{-1}(\frac{NA}{n}) \tag{1}$$

NA being the numerical aperture of the fiber and n the index of glass.
Polar coordinates were used to continue the analysis.

$$r^2 = x^2 + y^2 \tag{2}$$

A Gaussian model was used to calculate intensity:

$$I(r,z) = I_{max}(z)e^{\frac{-r^2}{2\sigma^2(z)}} \tag{3}$$

We wanted to calculate the parameters $I_{max}(z)$ and $\sigma(z)$
For $r = z\tan\alpha$, (On the diffraction cone of the fiber):

$$I(z\tan\alpha, z) = \frac{I_{max}(z)}{e} \tag{4}$$

Giving:

$$\sigma(z) = \frac{z\tan\alpha}{\sqrt{2}} \tag{5}$$

The power P at the end of the fiber was measured using a power meter. Therefore, if we sum the intensity on a plane it gives:

$$\int_{\varphi=0}^{2\pi}\int_{r=0}^{inf} I(r,z)rdrd\varphi = P \tag{6}$$

As a consequence:

$$I_{max}(z) = \frac{P}{\pi z^2 \tan^2\alpha} \tag{7}$$

In conclusion, the intensity can be expressed as:

$$I(r,z) = \frac{P}{\pi z^2 \tan^2 \alpha} e^{\frac{-r^2}{z^2 \tan^2 \alpha}} \quad (8)$$

<u>Intensity profile:</u>

We used formula 8 in a Matlab code to compute the intensity on different planes with different angle θ intersecting with point **P (See Supplementary Figure 1a)**. **Supplementary Figure 1b** shows that as the plane in which we computed the intensity (**Fig. S1a**, red line, perpendicular to the axis of the fiber) is tilted toward the plane between the glass and the brain (**Fig. S1a**, green line) the light beam enlarges. The intensity profile becomes elliptic. This effect decreases the average intensity of light. To take in account this decrease, we chose to express the average intensity as:

$$I_{mean} = \frac{P}{A_{1/e}} \quad (9)$$

Where $A_{1/e}$ represents the area where $I(r,z) > I_{max}^{plane}/e$, I_{max}^{plane} being the maximal intensity in the computed plane. $A_{1/e}$ is represented on **Supplementary Figure 1b** by the area inside of the white contour.

<u>Numerical Calculations:</u>

Two-photon imaging experiments: Experiments were performed on mice with cranial window made from a glass coverslip (100µm thick). The optic fiber ($NA = 0.275$) made an angle $\theta = 30°$ with the cranial window. **Fig. 2** shows that delivery of 473nm light at a power of 1mW, as measured at the tip of the fiber ($P = 1mW$), reliably triggered vasodilation. Therefore, from our calculations an average intensity of 25mW/mm² is enough to trigger a dilation of an arterial surface vessel. We did not observe any reliable vasodilation when the power measured at the tip of the fiber was reduced to 0.5mW and was delivered using the same protocol. Using this type of window and fiber configuration, we predict that the average light intensity sufficient to trigger arterial dilations at the brain surface is between 12.5mW/mm² and 25mW/mm².

fUS experiments: We used the same optic fiber as in the two-photon imaging experiments described above. The fiber made an angle $\theta = 30°$ with a PMP coverslip (250µm thick). **Fig. 1a** shows that using a measured power of 2mW at the tip of the fiber is sufficient to trigger an increase in the ultrasound Doppler signal. Using these parameters in our Matlab code, we find that the correspondent average intensity, at the surface of the brain, is 18mW/mm².
